# Effectiveness and safety of monoclonal antibodies against amyloid-beta *vis-à-vis* placebo in mild or moderate Alzheimer's disease

**DOI:** 10.3389/fneur.2023.1147757

**Published:** 2023-03-15

**Authors:** Ying Hao, Mingrui Dong, Yingtong Sun, Xiaohui Duan, Wenquan Niu

**Affiliations:** ^1^Institute of Clinical Medical Sciences, China-Japan Friendship Hospital, Beijing, China; ^2^Department of Neurology, China-Japan Friendship Hospital, Beijing, China

**Keywords:** Alzheimer's disease, monoclonal antibodies against amyloid-beta, effectiveness, adverse events, meta-analysis, MMSE, ADAS-Cog

## Abstract

**Backgrounds and objectives:**

Currently, no consensus has been reached on the therapeutic implications of monoclonal antibodies against amyloid-beta (Aβ) in Alzheimer's disease (AD). This study aimed to examine the effectiveness and safety of monoclonal antibodies against Aβ as a whole and also to determine the superiority of individual antibodies *vis-à-vis* placebo in mild or moderate AD.

**Methods:**

Literature retrieval, article selection, and data abstraction were performed independently and in duplicate. Cognition and function were appraised by the Mini-Mental State Examination (MMSE), Alzheimer's Disease Assessment Scale-Cognitive Subscale (ADAS-Cog), Disability Assessment for Dementia (DAD), and Clinical Dementia Rating Scale-Sum of Boxes (CDR-SB). Effect sizes are expressed as standardized mean difference (SMD) with a 95% confidence interval (CI).

**Results:**

Twenty-nine articles involving 108 drug-specific trials and 21,383 participants were eligible for synthesis. Of the four assessment scales, only CDR-SB was significantly reduced after using monoclonal antibodies against Aβ relative to placebo (SMD: −0.12; 95% CI: −0.2 to −0.03; *p* = 0.008). Egger's tests indicated a low likelihood of publication bias. At individual levels, bapineuzumab was associated with a significant increase in MMSE (SMD: 0.588; 95% CI: 0.226–0.95) and DAD (SMD: 0.919; 95% CI: 0.105–1.943), and a significant decrease in CDR-SB (SMD: −0.15; 95% CI: −0.282–0.018). Bapineuzumab can increase the significant risk of serious adverse events (OR: 1.281; 95% CI: 1.075–1.525).

**Conclusion:**

Our findings indicate that monoclonal antibodies against Aβ can effectively improve instrumental activities of daily life in mild or moderate AD. In particular, bapineuzumab can improve cognition and function, as well as activities of daily life, and meanwhile, it triggers serious adverse events.

## Introduction

Alzheimer's disease (AD) is a chronic neurodegenerative disease with insidious clinical presentation, and it is characterized by progressive impairment of memory and cognitive function. Approximately 50 million people are suffering from dementia globally, and the number elevates by 10 million annually, as per the 2020 report of the World Health Organization (WHO) (1). By 2050, the cases of dementia are expected to triple (2). As revealed by a systematical analysis in 2020, the overall prevalence of AD was 3.2% in Chinese individuals over 60 years, and its annual prevalence was predicted to increase from 3.81 to 6.17% within the next 5 years (3). In India, the incidence rate of AD per 1,000 person-years was 11.67 for those aged ≥ 55 years (4). Patients diagnosed with AD often experience slow and variable clinical courses, and their original survival ability gradually decreases, eventually leading to death due to complications (5). It is, hence, clinically meaningful to retard, prevent, or even reverse neurological and functional impairment through early and effective pharmacologic treatment.

Alzheimer's disease is a multifactorial disorder involving interactions among genetic, environmental, and lifestyle factors, which open new avenues for the development of tailored therapeutics in the era of precision medicine (6). It is widely recognized that dementia is the underlying cause of AD, and it accounts for 60% of cases (7). AD progresses rapidly, yet treatment options are very limited. Some approved drugs targeting AD, such as donepezil, galantamine, rivastigmine, and memantine, can only help relieve patients' symptoms and suppress the psychological and behavioral symptoms of dementia. Several theories existed for the pathophysiology of AD, including the amyloid cascade hypothesis, degeneration of neuronal cells, and aggregation of tau proteins within the cell (8, 9). Thereof, the amyloid cascade hypothesis is widely accepted, and it proposes that the neurodegeneration and resultant dementia of AD occur as a result of the formation and accumulation of toxic, soluble amyloid-beta (Aβ) oligomers, formed by the misfolding of Aβ monomers (10). In the literature, different therapeutic strategies to clear Aβ from the brain were developed, and monoclonal antibodies against amyloid-beta (Aβ) have aroused growing concerns (11). There is clinical evidence that immunotherapy with monoclonal antibodies is effective for the treatment of patients at earlier AD stages before the emergence of dementia (12). Bapineuzumab is the first N-terminus-directed anti-Aβ antibody tested in humans. Subsequently, several anti-Aβ monoclonal antibody drugs were tested by clinical trials (13). Moreover, aducanumab, a human Ig monoclonal antibody, is recognized as being “risen from the grave,” and it acts in Aβ clearance and curtailing calcium defects in AD (14). Other treatment potentials, such as the immune response generating active immunotherapy and passive immunotherapeutic approaches targeting monoclonal antibodies toward Aβ aggregates, were also proposed (10). Of all anti-Aβ regimens, passive immunization with anti-Aβ antibodies is recognized as being safe and well-tolerated (15), whereas no consensus has been reached upon the therapeutic implications of monoclonal antibodies against Aβ in AD. Fortunately, meta-analysis can provide an opportunity to help derive more reliable estimates.

We aimed to examine the effectiveness and safety profiles of monoclonal antibodies against Aβ as a whole and also to determine the superiority of individual monoclonal antibodies against Aβ *vis-à-vis* placebo in the treatment of patients with mild or moderate AD.

## Methods

### Guidelines

The conduct of this meta-analysis conformed to the statement in the Preferred Reporting Items for Systematic Reviews and Meta-Analyses (PRISMA) guidelines (16). The PRISMA checklist is provided in Supplementary Table 1.

### Search strategy

Potential clinical trials were searched from PubMed, Excerpta Medica Database (EMBASE), and Web of Science, and the last search was conducted on 31 March 2022. The keywords used for the literature search are expressed in the Boolean form, that is (Alzheimer's^*^ OR dementia^*^), in the Title/Abstract AND (Aducanumab^*^ OR aduhelm OR BIIB-037 OR BIIB037 OR Solanezumab^*^ OR LY 2062430 OR LY2062430 OR LY-2062430 OR Bapineuzumab^*^ OR AAB-001 OR AAB 001 OR Gantenerumab^*^ OR RG-1450 OR R-1450 OR R1450 OR RG1450 OR R04909832 OR R-04909832 OR RO-4909832 OR Crenezumab^*^ OR MABT5102A OR MABT-5102A OR RG7412 OR RG-7412 OR Ponezumab^*^ OR RN-1219 OR PF-04360365) in the Title/Abstract AND (clinical AND trial OR random^*^) in the Title/Abstract. In addition, the bibliographies of identified trials were scanned for additional references. All trials were conducted in humans and reported in English. Trials were searched independently by two authors (Y.H. and M.D.), and any disagreement was resolved by discussion with a third author (W.N.).

### Inclusion/exclusion criteria

Trials were eligible for inclusion if they met the following criteria simultaneously: (i) participants: patients with mild or moderate AD; (ii) intervention: monoclonal antibodies against Aβ and placebo; (iii) comparator: control; (iv) clinical outcomes: changes in one of the four scales adopted to assess the cognition and function aspects of AD, including the Mini-Mental State Examination (MMSE), Alzheimer's Disease Assessment Scale-Cognitive Subscale (ADAS-Cog), Disability Assessment for Dementia (DAD), and Clinical Dementia Rating Scale-Sum of Boxes (CDR-SB); (v) study design: randomized controlled trials; and (vi) formal publication in peer-review journals.

Trials were excluded if one or more of the following criteria were satisfied: (i) publication type: narrative or systematic review, meta-analysis, case report, case series, conference abstract, comment, correspondence, or editorial; (ii) duplication publication; (iii) lack of comparator; (iv) control rather than placebo; and (v) clinical outcomes rather than four assessment scales mentioned earlier. In the case of more than one article was published using the overlapped study participants, the article with the largest sample size was retained in this meta-analysis.

The eligibility assessment of each retrieved trial was made by two authors (Y.H. and M.D.) independently. Any discrepancy was solved by discussion, and if necessary, was adjudicated by a third author (W.N.).

### Data collection

Data from each qualified article were separately abstracted from each qualified article by two reviewers (Y.H. and M.D.) and were typed into a predesigned Excel file, including the surname of the first author, year of publication, ethnicity, and country where participants were enrolled, study design, trial phase, intervention drugs and doses, degree of AD, intervention period, sample size of each arm, number of responses, and dropouts during regimen treatment, and assessment scales for AD, as well as some baseline characteristics, including age, gender, weight, height, body mass index, duration of AD, use of AChEI (acetylcholinesterase inhibitors) or memantine, four assessment scales associated with the risk of AD and adverse reactions, when available.

The process of data collection was completed independently and in duplicate (Y.H. and M.D.), and the consistency of the two datasets was tested by the kappa statistic. In the case of kappa statistics less than unity, original data were checked, and if necessary, a third author (W.N.) was involved.

### Quality assessment

Risk of bias for each clinical trial was assessed using the “Revised Cochrane risk-of-bias tool for randomized trials” (RoB 2) (17) from the following five aspects, that is, randomization process, bias due to deviations of intended interventions, bias due to missing outcome data, bias in outcome measurements, and bias in the selection of reported results. Individual domains of risk of bias can be categorized as “low risk,” “some concerns,” or “high risk.” Quality assessment was performed by two authors (Y.H. and M.D.), and any disagreement was solved by a third author (W.N.).

### Statistical analyses

Data were imported from Excel to STATA software version 16 (Stata Corp, College Station, Texas, USA), which was used to handle statistical analyses in this meta-analysis. Effect-size estimates from individual trials were pooled under random-effect models, irrespective of the presence or absence of statistical heterogeneity across trials (18). Statistical heterogeneity was measured by the *I*^2^ metric, which ranges from 0 to 100%, with higher values representing greater degree of heterogeneity.

The changes in assessment scales for AD before and after intervention are expressed as a standardized mean difference (SMD) with a 95% confidence interval (95% CI) because different rating subscales were used, and the changes in adverse events after intervention are expressed as odds ratio (OR) with a 95% CI.

Cumulative analyses were used to measure the influence of first published trials on subsequent publications and the evolution of accumulated estimates over time. Sensitivity analyses were used to assess the influence of any single trial on pooled effect-size estimates by removing one trial at a time.

Publication bias was inspected using Begg's funnel plots and Egger's tests. The significance of Egger's tests was set at 10%. In addition, to yield more information, the Duval and Tweedie non-parametric “trim and fill” method was employed to estimate the number of theoretically missing trials and derive “unbiased” effect-size estimates.

## Results

### Eligible articles

**Figure 1:**
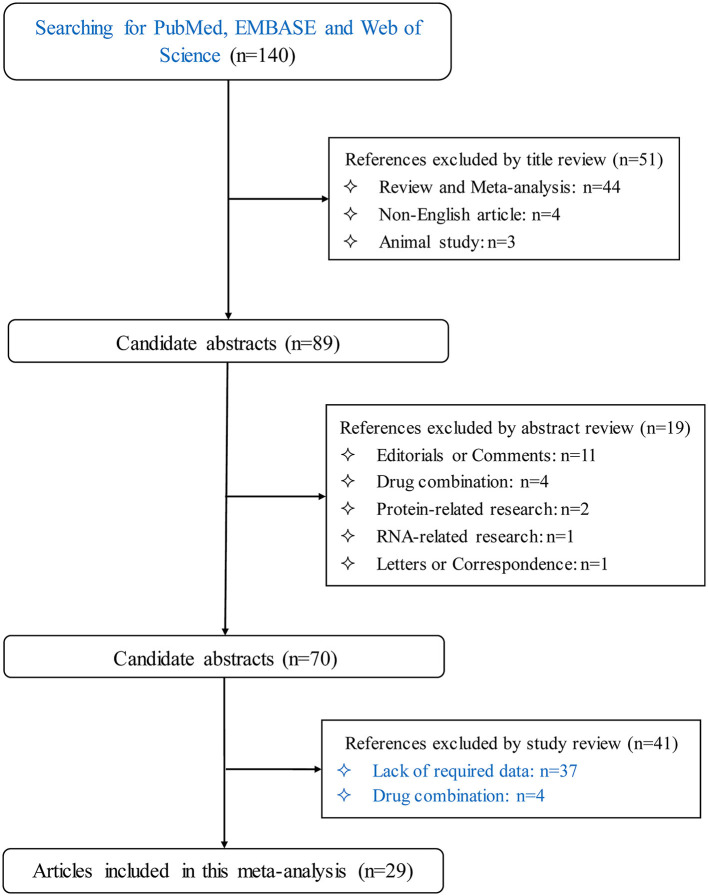
PRISMA flowchart illustrates the selection process of qualified articles with specific reasons for exclusion in this meta-analysis.

By using the prespecified key terms, the literature search of three public databases retrieved a total of 140 publications. After applying predesigned inclusion and exclusion criteria, only 29 articles published in English from 2009 to 2021 were eligible for the final analysis (19–47), involving 108 drug-specific trials and 21,383 participants. Figure 1 illustrates the process of article selection for this meta-analysis.

### Trial characteristics

Table 1 shows the trial characteristics in this meta-analysis. Five trials involved patients with mild AD, 95 trials involved patients with mild or moderate AD, and five trials involved patients with prodromal or mild AD. Forty-six trials were in phase I, 26 in phase II, two in phase II–III, 26 in phase III, and eight in unreported phases. Trial duration ranged from 12 to 208 weeks. In terms of risk of bias, all clinical trials involved in this meta-analysis were classified as “low risk” or “having some concerns” due to missing necessary information.

**Table 1:** Characteristics of eligible trials evaluated in this meta-analysis.

**First author**	**Year**	**Country**	**Race**	**Drug**	**Dose**	**Clinical phase**	**Design**	**AD stage**	**Study duration (weeks)**
Salloway (Bapi 0.15–2)	2009	USA	White	Bapineuzumab	0.15–2	II	Double-blinded	Mild or moderate	78
Salloway (Bapi 0.15)	2009	USA	White	Bapineuzumab	0.15	II	Double-blinded	Mild or moderate	78
Salloway (Bapi 0.5)	2009	USA	White	Bapineuzumab	0.5	II	Double-blinded	Mild or moderate	78
Salloway (Bapi 1)	2009	USA	White	Bapineuzumab	1	II	Double-blinded	Mild or moderate	78
Salloway (Bapi 2)	2009	USA	White	Bapineuzumab	2	II	Double-blinded	Mild or moderate	78
Black (Bapi 0.5)	2010	USA	White	Bapineuzumab	0.5	I	Unblinded	Mild or moderate	52
Black (Bapi 1.5)	2010	USA	White	Bapineuzumab	1.5	I	Unblinded	Mild or moderate	52
Black (Bapi 5)	2010	USA	White	Bapineuzumab	5	I	Unblinded	Mild or moderate	52
Rinne (Bapi 0.5–2)	2010	UK/Finland	White	Bapineuzumab	0.15–2.0	II	Double-blinded	Mild or moderate	78
Salloway (Bapi 0.5 in ε4 carriers)	2014	USA	White	Bapineuzumab	0.5	III	Double-blinded	Mild or moderate	78
Salloway (Bapi 0.5 in ε4 non-carriers)	2014	USA	White	Bapineuzumab	0.5	III	Double-blinded	Mild or moderate	78
Salloway (Bapi 1.0 in ε4 non-carriers)	2014	USA	White	Bapineuzumab	1	III	Double-blinded	Mild or moderate	78
Arai (Bapi 0.15)	2016	Japan	Japanese	Bapineuzumab	0.15	I	Double-blinded	Mild or moderate	52
Arai (Bapi 0.5)	2016	Japan	Japanese	Bapineuzumab	0.5	I	Double-blinded	Mild or moderate	52
Arai (Bapi 1)	2016	Japan	Japanese	Bapineuzumab	1	I	Double-blinded	Mild or moderate	52
Arai (Bapi 2.0)	2016	Japan	Japanese	Bapineuzumab	2	I	Double-blinded	Mild or moderate	52
Brody (Bapi 2)	2016	USA	White	Bapineuzumab	0.03	II	Double-blinded	Mild or moderate	52
Brody (Bapi 7)	2016	USA	White	Bapineuzumab	0.1	II	Double-blinded	Mild or moderate	52
Brody (Bapi 20)	2016	USA	White	Bapineuzumab	0.3	II	Double-blinded	Mild or moderate	52
Ivanoiu (Bapi 0.5 in ε4 carriers)	2016	Europe-USA	White	Bapineuzumab	0.5	III	Double-blinded	Mild or moderate	78
Ivanoiu (Bapi 0.5 in ε4 non-carriers)	2016	Europe-USA	White	Bapineuzumab	0.5	III	Double-blinded	Mild or moderate	78
Ivanoiu (Bapi 1.0 in ε4 non-carriers)	2016	Europe-USA	White	Bapineuzumab	1	III	Double-blinded	Mild or moderate	78
Vandenberghe (Bapi 0.5 in ε4 carriers)	2016	Belgium	White	Bapineuzumab	0.5	III	Double-blinded	Mild or moderate	78
Vandenberghe (Bapi 0.5 in ε4 non-carriers)	2016	Belgium	White	Bapineuzumab	0.5	III	Double-blinded	Mild or moderate	78
Vandenberghe (Bapi 1.0 in ε4 non-carriers)	2016	Belgium	White	Bapineuzumab	1	III	Double-blinded	Mild or moderate	78
Brashear (Bapi 0.5 in ε4 carriers)	2018	USA/Canada/Germany/Austria	White	Bapineuzumab	0.5	III	Double-blinded	Mild or moderate	83
Brashear (Bapi 0.5 in ε4 non-carriers)	2018	USA/Canada/Germany/Austria	White	Bapineuzumab	0.5	III	Double-blinded	Mild or moderate	83
Brashear (Bapi 1.0 in ε4 non-carriers)	2018	USA/Canada/Germany/Austria	White	Bapineuzumab	1	III	Double-blinded	Mild or moderate	83
Brashear (Bapi 2.0 in ε4 non-carriers)	2018	USA/Canada/Germany/Austria	White	Bapineuzumab	2	III	Double-blinded	Mild or moderate	83
Lu (Bapi 5–80)	2019	USA	White	Bapineuzumab	0.07–1.2	I	Double-blinded	Mild or moderate	17
Lu (Bapi 5)	2019	USA	White	Bapineuzumab	0.07	I	Double-blinded	Mild or moderate	17
Lu (Bapi 10)	2019	USA	White	Bapineuzumab	0.14	I	Double-blinded	Mild or moderate	17
Lu (Bapi 20)	2019	USA	White	Bapineuzumab	0.3	I	Double-blinded	Mild or moderate	17
Lu (Bapi 40)	2019	USA	White	Bapineuzumab	0.6	I	DOUBLE-blinded	Mild or moderate	17
Lu (Bapi 80)	2019	USA	White	Bapineuzumab	1.2	I	Double-blinded	Mild or moderate	17
Delnomdedieu (AAB-003 0.5)	2016	Korea/USA	White	Bapineuzumab_modified	0.5	I	Double-blinded	Mild or moderate	52
Delnomdedieu (AAB-003 1)	2016	Korea/USA	White	Bapineuzumab_modified	1	I	Double-blinded	Mild or moderate	52
Delnomdedieu (AAB-003 2)	2016	Korea/USA	Asian	Bapineuzumab_modified	2	I	Double-blinded	Mild or moderate	52
Delnomdedieu (AAB-003 4)	2016	Korea/USA	Asian	Bapineuzumab_modified	4	I	Double-blinded	Mild or moderate	52
Delnomdedieu (AAB-003 8)	2016	Korea/USA	White	Bapineuzumab_modified	8	I	Double-blinded	Mild or moderate	52
Ferrero (Aduc 0.3–60)	2016	USA	White	Aducanumab	0.3–60	I	Double-blinded	Mild or moderate	26
Ferrero (Aduc 0.3)	2016	USA	White	Aducanumab	0.3	I	Double-blinded	Mild or moderate	26
Ferrero (Aduc 1)	2016	USA	White	Aducanumab	1	I	Double-blinded	Mild or moderate	26
Ferrero (Aduc 3)	2016	USA	White	Aducanumab	3	I	Double-blinded	Mild or moderate	26
Ferrero (Aduc 10)	2016	USA	White	Aducanumab	10	I	Double-blinded	Mild or moderate	26
Ferrero (Aduc 20)	2016	USA	White	Aducanumab	20	I	Double-blinded	Mild or moderate	26
Ferrero (Aduc 30)	2016	USA	White	Aducanumab	30	I	Double-blinded	Mild or moderate	26
Ferrero (Aduc 60)	2016	USA	White	Aducanumab	60	I	Double-blinded	Mild or moderate	26
Sevigny (Aduc 1.0–10)	2016	USA	Others	Aducanumab	1.0–10.0	III	Double-blinded	Prodromal or mild	52
Sevigny (Aduc 1)	2016	USA	Others	Aducanumab	1	III	Double-blinded	Prodromal or mild	52
Sevigny (Aduc 3)	2016	USA	Others	Aducanumab	3	III	Double-blinded	Prodromal or mild	52
Sevigny (Aduc 6)	2016	USA	Others	Aducanumab	6	III	Double-blinded	Prodromal or mild	52
Sevigny (Aduc 10)	2016	USA	Others	Aducanumab	10	III	Double-blinded	Prodromal or mild	52
Siemers (Sola 0.5)	2010	Indiana	Others	Solanezumab	0.5	I	Double-blinded	Mild or moderate	52
Siemers (Sola 1.5)	2010	Indiana	Others	Solanezumab	1.5	I	Double-blinded	Mild or moderate	52
Siemers (Sola 4)	2010	Indiana	Others	Solanezumab	4	I	Double-blinded	Mild or moderate	52
Siemers (Sola 10)	2010	Indiana	Others	Solanezumab	10	I	Double-blinded	Mild or moderate	52
Farlow (LY100 mg Q4W)	2012	USA	White	Solanezumab	0.4	II	Double-blinded	Mild or moderate	12
Farlow (LY100 mg QW)	2012	USA	White	Solanezumab	1.4	II	Double-blinded	Mild or moderate	12
Farlow (LY400 mg Q4W)	2012	USA	White	Solanezumab	1.5	II	Double-blinded	Mild or moderate	12
Farlow (LY400 mg QW)	2012	USA	White	Solanezumab	5.8	II	Double-blinded	Mild or moderate	12
Uenaka (Sola 0.5)	2012	Japan	Japanese	Solanezumab	0.5	NA	Double-blinded	Mild or moderate	16
Uenaka (Sola 1.5)	2012	Japan	Japanese	Solanezumab	1.5	NA	Double-blinded	Mild or moderate	16
Uenaka (Sola 4)	2012	Japan	Japanese	Solanezumab	4	NA	Double-blinded	Mild or moderate	16
Uenaka (Sola 10)	2012	Japan	Japanese	Solanezumab	10	NA	Double-blinded	Mild or moderate	16
Uenaka (Sola 0.5)	2012	USA	White	Solanezumab	0.5	NA	Single-blinded	Mild or moderate	16
Uenaka (Sola 1.5)	2012	USA	White	Solanezumab	1.5	NA	Single-blinded	Mild or moderate	16
Uenaka (Sola 4)	2012	USA	White	Solanezumab	4	NA	Single-blinded	Mild or moderate	16
Uenaka (Sola 10)	2012	USA	White	Solanezumab	10	NA	Single-blinded	Mild or moderate	16
Doody (All)	2014	Multiple countries	White	Solanezumab	5.8	III	Double-blinded	Mild or moderate	78
Doody (Sola E1 in mild or moderate)	2014	Multiple countries	White	Solanezumab	5.8	III	Double-blinded	Mild or moderate	78
Doody (Sola E2 in all)	2014	Multiple countries	White	Solanezumab	5.8	III	Double-blinded	Mild or moderate	78
Doody (Sola E2 in mild)	2014	Multiple countries	White	Solanezumab	5.8	III	Double-blinded	Mild	78
Siemers (Sola)	2016	Multiple countries	White	Solanezumab	5.8	III	Double-blinded	Mild	80
Honig (Sola)	2018	USA	White	Solanezumab	5.8	III	Double-blinded	Mild	80
Salloway (Gant)	2021	Multiple countries	Others	Gantenerumab	3.2–17.3	II-III	Double-blinded	Mild	208
Salloway (Sola)	2021	Multiple countries	Others	Solanezumab	5.8–23.1	II-III	Double-blinded	Mild	208
Ostrowitzki (Gant 105)	2017	Europe	Others	Gantenerumab	8.1	III	Double-blinded	Mild or moderate	104
Ostrowitzki (Gant 225)	2017	Europe	Others	Gantenerumab	18.6	III	Double-blinded	Mild or moderate	104
Cummings (Cren-low)	2018	North America/Europe	Others	Crenezumab	4	II	Double-blinded	Mild or moderate	76
Cummings (Cren-high)	2018	North America/Europe	Others	Crenezumab	15	II	Double-blinded	Mild or moderate	76
Salloway (Cren-both)	2018	USA/France/Spain	Others	Crenezumab		II	Double-blinded	Mild or moderate	73
Salloway (Cren-low)	2018	USA/France/Spain	Others	Crenezumab	4.3	II	Double-blinded	Mild or moderate	73
Salloway (Cren-high)	2018	USA/France/Spain	Others	Crenezumab	15	II	Double-blinded	Mild or moderate	73
Guthrie (Cren 120)	2020	USA	White	Crenezumab	120	I	Double-blinded	Mild or moderate	13
Guthrie (Cren 30)	2020	USA	White	Crenezumab	30	I	Double-blinded	Mild or moderate	13
Guthrie (Cren 45)	2020	USA	White	Crenezumab	45	I	Double-blinded	Mild or moderate	13
Guthrie (Cren 60)	2020	USA	White	Crenezumab	60	I	Double-blinded	Mild or moderate	13
Landen (Pone 0.1–10)	2013	Canada/Australia/United Kingdom	White	Ponezumab	0.1–10.0	I	Double-blinded	Mild or moderate	52
Landen (Pone 0.1)	2013	Canada/Australia/United Kingdom	White	Ponezumab	0.1	I	Double-blinded	Mild or moderate	52
Landen (Pone 0.3)	2013	Canada/Australia/United Kingdom	White	Ponezumab	0.3	I	Double-blinded	Mild or moderate	52
Landen (Pone 1)	2013	Canada/Australia/United Kingdom	White	Ponezumab	1	I	Double-blinded	Mild or moderate	52
Landen (Pone 3)	2013	Canada/Australia/United Kingdom	White	Ponezumab	3	I	Double-blinded	Mild or moderate	52
Landen (Pone 10)	2013	Canada/Australia/United Kingdom	White	Ponezumab	10	I	Double-blinded	Mild or moderate	52
Landen (Pone 10)	2017	Sweden	White	Ponezumab	10	II	Double-blinded	Mild	52
Landen (Pone 7.5)	2017	Sweden	White	Ponezumab	7.5	II	Double-blinded	Mild	52
Landen (Pone 0.1)	2017	Western countries/Japan	White	Ponezumab	0.1	II	Double-blinded	Mild or moderate	78
Landen (Pone 0.5)	2017	Western countries/Japan	White	Ponezumab	0.5	II	Double-blinded	Mild or moderate	78
Landen (Pone 1.0)	2017	Western countries/Japan	White	Ponezumab	1	II	Double-blinded	Mild or moderate	78
Landen (Pone 3)	2017	Western countries/Japan	White	Ponezumab	3	II	Double-blinded	Mild or moderate	78
Landen (Pone 8.5)	2017	Western countries/Japan	White	Ponezumab	8.5	II	Double-blinded	Mild or moderate	78
Lowe (Dona SD 10)	2021	USA/Japan	White	Donanemab	10	I	Double-blinded	Mild or moderate	72
Lowe (Dona SD 20)	2021	USA/Japan	White	Donanemab	20	I	Double-blinded	Mild or moderate	72
Lowe (Dona SD 40)	2021	USA/Japan	White	Donanemab	40	I	Double-blinded	Mild or moderate	72
Lowe (Dona Q2W 10)	2021	USA/Japan	White	Donanemab	5	I	Double-blinded	Mild or moderate	72
Lowe (Dona Q4W 10)	2021	USA/Japan	White	Donanemab	2.5	I	Double-blinded	Mild or moderate	72
Lowe (Dona Q4W 20)	2021	USA/Japan	White	Donanemab	5	I	Double-blinded	Mild or moderate	72
Mintun (Dona)	2021	USA/Canada	White	Donanemab	10.1–20.2	II	Double-blind	Mild	72

### Monoclonal antibodies against Aβ

Table 2 shows the detailed targeting information of monoclonal antibodies against Aβ under evaluation. Specifically, eight monoclonal antibodies against Aβ were available, including aducanumab (BIIB037), bapineuzumab (AAB-001), bapineuzumab modified (AAB-003), crenezumab (MABT5102A), donanemab (LY3002813), gantenerumab (R1450/RO4909832), ponezumab (PF04360365), and solanezumab (LY100/LY2062430). Comparison with placebo was available for aducanumab in 13 trials, for bapineuzumab in 35 trials, for bapineuzumab modified in five trials, for crenezumab in nine trials, for donanemab in seven trials, for gantenerumab in three trials, for ponezumab in 13 trials, and for solanezumab in 23 trials.

**Table 2:** Major monoclonal antibody immune drugs.

**Monoclonal antibodies against Aβ**	**Alias**	**Antibody type**	**Epitope**	**Clinical stage**
Aducanumab	BIIB037	IgG1	Aβ (aa 3–6)	III
Bapineuzumab	AAB-001	IgG1	Aβ (aa 1–5)	III
Bapineuzumab-modified	AAB-003	IgG1	Aβ (aa 1–5)	I
Crenezumab	MABT5102A	IgG4	Aβ (aa 13–24)	II
Donanemab	LY3002813	IgG1	Aβ (p 3–42)	II
Gantenerumab	R1450/RO4909832	IgG1	Aβ (aa 1–5) and Aβ (aa 18–27)	III
Ponezumab	PF04360365	IgG2	Aβ (aa 30–40)	II
Solanezumab	LY100/LY2062430	IgG1	Aβ (aa 16–26)	III

### Overall estimation

**Figure 2:**
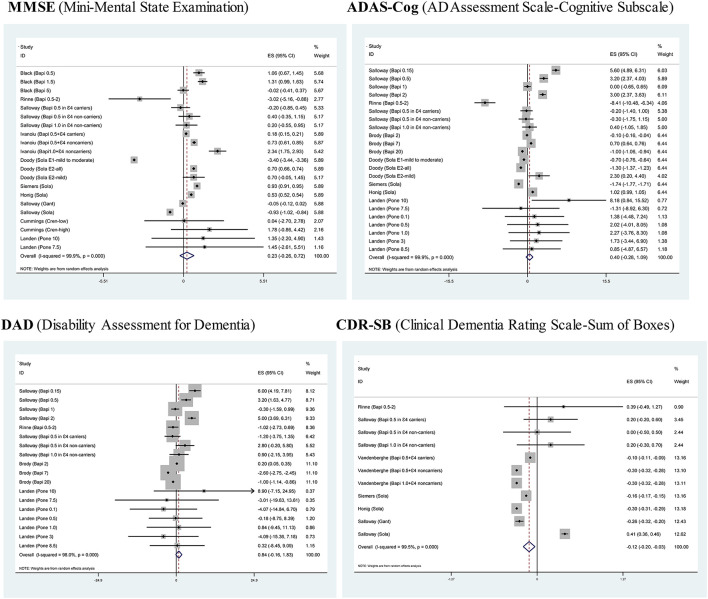
Forest plots of four assessment scales for monoclonal antibodies against Aβ *vis-à-vis* placebo in the treatment of mild or moderate Alzheimer's disease.

Figure 2 provides the forest plots of four assessment scales for monoclonal antibodies against Aβ *vis-à-vis* placebo in the treatment of AD. Of four assessment scales, only CDR-SB was significantly reduced after using monoclonal antibodies against Aβ relative to placebo (SMD: −0.12; 95% CI: −0.2 to −0.03; *p* = 0.008), indicating that monoclonal antibodies against Aβ can effectively improve instrumental activities of daily life. Statistical heterogeneity across trials for each assessment scale was significant (*I*^2^ > 90%; *p* < 0.001).

### Cumulative and influential analyses

Supplementary Figures 1, 2 separately show the cumulative and influential analyses of four assessment scales for monoclonal antibodies against Aβ *vis-à-vis* placebo in the treatment of AD.

### Publication bias

**Figure 3:**
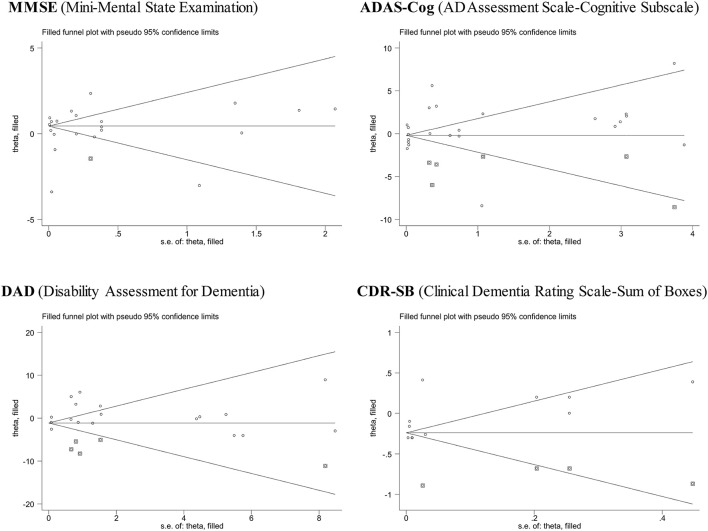
Funnel plots of four assessment scales for monoclonal antibodies against Aβ *vis-à-vis* placebo in the treatment of mild or moderate Alzheimer's disease.

Figure 3 presents the filled funnel plots of four assessment scales for monoclonal antibodies against Aβ *vis-à-vis* placebo in the treatment of AD. There were separately one, six, five, and four theoretically missing studies required to make the funnel plots symmetrical for MMSE, ADAS-Cog, DAD, and CDR-SB. Egger's test indicated a low likelihood of publication bias, with the corresponding probabilities being 0.687, 0.434, 0.880, and 0.282.

### Subsidiary estimation

As different monoclonal antibodies against Aβ might exert a diverse impact on assessment scales, drug-specific subsidiary analyses were done accordingly (Table 3). To control potential bias from small-scale estimation, only subgroups involving three or more trials are displayed. Specifically, bapineuzumab was associated with a significant increase in MMSE (SMD: 0.588; 95% CI: 0.226–0.95) and DAD (SMD: 0.919; 95% CI: 0.105–1.943), while a significant decrease in CDR-SB (SMD: −0.15; 95% CI: −0.282–0.018), indicating that bapineuzumab can not only improve cognitive outcomes and functional abilities but also instrumental activities of daily life.

**Table 3:** Drug-specific comparisons of four assessment scales associated with mild or moderate AD.

**Monoclonal antibodies against Aβ**	**Studies (*n*)**	**SMD (95% CI)**	***P***	***I* ^2^**	***P* for heterogeneity**
**MMSE**					
Bapineuzumab	10	0.588 (0.226 to 0.950)	0.001	95.57%	<0.001
**ADAS-COG**					
Bapineuzumab	11	0.675 (0.048 to 1.302)	0.035	99.50%	<0.001
Ponezumab	7	2.012 (−0.294 to 4.318)	0.087	0.00%	0.722
Solanezumab	5	−0.235 (−1.722 to 1.253)	0.757	99.98%	<0.001
**DAD**					
Bapineuzumab	11	0.919 (0.105 to 1.943)	<0.001	98.84%	<0.001
Ponezumab	7	−0.566 (−4.646 to 0.514)	0.786	0.00%	0.888
**CDR-SB**					
Bapineuzumab	7	−0.15 (−0.282 to 0.018)	0.026	98.92%	<0.001
Solanezumab	3	−0.021 (−0.183 to 0.141)	0.799	99.84%	<0.001

MMSE, Mini-Mental State Examination; ADAS-Cog, Cognitive Subscale; DAD, Disability Assessment for Dementia; CDR-SB, Clinical Dementia Rating Scale-Sum of Boxes; Aβ, amyloid-beta; SMD, standardized mean difference; 95% CI, 95% confidence interval.

In addition, it is surprising to note that bapineuzumab can significantly increase ADAS-Cog (SMD: 0.675; 95% CI: 0.048–1.302). Changes in the four scores were not significant for the other types of monoclonal antibodies against Aβ.

### Adverse events

Table 4 summarizes the common adverse events associated with monoclonal antibodies against Aβ *vis-à-vis* placebo in the treatment of AD. Relative to the other types of monoclonal antibodies against Aβ, bapineuzumab can increase the significant risk of serious adverse events (OR: 1.281; 95% CI: 1.075–1.525) during the treatment of patients with mild or moderate AD. As for donanemab, there was a significantly increased risk of urinary tract infection (OR: 2.452; 95% CI: 1.107–5.428), nervous system disorders (OR: 3.368; 95% CI: 1.49–7.612), intracranial hemorrhage (OR: 4.966; 95% CI: 1.68–10.674), and amyloid-related imaging abnormalities (OR: 3.063; 95% CI: 3.525–23.3).

**Table 4:** Common adverse events associated with monoclonal antibodies against Aβ in the treatment of mild or moderate AD.

**Adverse events**	**Studies (*n*)**	**OR (95% CI)**	***P***	***I* ^2^**	***P* for heterogeneity**
**Serious adverse events**					
Bapineuzumab	20	1.281 (1.075 to 1.525)	0.006	13.50%	<0.001
Crenezumab	5	1.377 (0.838 to 2.263)	0.206	0%	0.97
Donanemab	7	0.916 (0.536 to 1.565)	0.748	0%	0.992
Ponezumab	6	2.845 (0.913 to 8.861)	0.071	0%	0.999
Solanezumab	5	0.961 (0.815 to 1.133)	0.633	0%	0.721
**Treatment emergent adverse event**					
Bapineuzumab	8	1.181 (0.949 to 1.470)	0.137	0%	0.564
Donanemab	6	1.384 (0.506 to 3.786)	0.527	0%	0.964
**Vertigo**					
Bapineuzumab	6	1.165 (0.361 to 3.762)	0.799	0%	0.997
Solanezumab	5	0.667 (0.152 to 2.915)	0.59	0%	0.999
**Diarrhea**					
Bapineuzumab	6	1.185 (0.894 to 1.572)	0.238	0%	0.938
Ponezumab	5	1.187 (0.309 to 4.561)	0.803	49.07%	0.097
Solanezumab	6	0.986 (0.781 to 1.244)	0.903	0%	0.889
**Nausea**					
Bapineuzumab	6	0.932 (0.669 to 1.299)	0.678	0%	0.703
Bapineuzumab modified	5	1.907 (0.546 to 6.664)	0.312	0%	0.962
Ponezumab	5	3.121 (1.078 to 9.034)	0.036	0%	0.931
Solanezumab	7	1.083 (0.819 to 1.433)	0.576	0%	0.971
**Vomiting**					
Bapineuzumab	10	0.996 (0.666 to 1.491)	0.986	0%	0.78
Bapineuzumab modified	5	1.907 (0.546 to 6.664)	0.312	0%	0.962
Donanemab	7	2.675 (1.111 to 6.444)	0.028	0%	0.999
Solanezumab	7	1.114 (0.82 to 1.512)	0.49	0%	0.599
**Constipation**					
Bapineuzumab	3	1.343 (0.794 to 2.27)	0.271	0%	0.853
Ponezumab	5	2.698 (0.931 to 7.822)	0.068	0%	0.996
**Fatigue**					
Bapineuzumab	6	0.888 (0.393 to 2.005)	0.774	0%	0.986
Donanemab	6	2.57 (0.798 to 8.278)	0.114	0%	0.996
Ponezumab	5	2.258 (0.962 to 5.299)	0.061	0%	0.776
Solanezumab	10	1.058 (0.786 to 1.424)	0.71	0%	0.98
**By infections**					
Bapineuzumab	7	1.159 (0.726 to 1.851)	0.537	0%	0.907
**Nasopharyngitis**					
Bapineuzumab	5	1.127 (0.704 to 1.803)	0.619	0%	0.975
Bapineuzumab modified	5	1.901 (0.546 to 6.664)	0.312	0%	0.962
Crenezumab	5	0.607 (0.259 to 1.424)	0.251	54.61%	0.066
Gantenerumab	3	1.566 (1.036 to 2.365)	0.033	0%	0.653
Ponezumab	5	1.346 (0.574 to 3.155)	0.494	0%	0.786
Solanezumab	12	1.022 (0.821 to 1.273)	0.846	0%	0.834
**Urinary tract infection**					
Bapineuzumab	6	1.044 (0.797 to 1.367)	0.757	0%	0.974
Bapineuzumab modified	5	1.907 (0.546 to 6.664)	0.312	0%	0.962
Crenezumab	2	1.046 (0.554 to 1.977)	0.889	0%	0.444
Donanemab	7	2.452 (1.107 to 5.428)	0.027	0%	0.999
Ponezumab	5	1.14 (0.513 to 2.532)	0.748	0%	0.91
Solanezumab	8	0.825 (0.655 to 1.039)	0.101	0%	0.991
**Upper respiratory tract infection**					
Aducanumab	8	0.404 (0.129 to 1.265)	0.12	0%	0.956
Bapineuzumab	12	0.872 (0.581 to 1.308)	0.507	0%	0.495
Crenezumab	5	1.115 (0.62 to 2.004)	0.716	0%	0.438
Donanemab	7	0.852 (0.431 to 1.686)	0.646	0%	0.986
Ponezumab	5	0.649 (0.318 to 1.324)	0.234	0%	0.87
Solanezumab	6	0.864 (0.62 to 1.204)	0.388	11.17%	0.344
**Pneumonia**					
Bapineuzumab	7	1.117 (0.591 to 2.111)	0.733	0%	0.95
Crenezumab	5	0.835 (0.263 to 2.653)	0.76	0%	0.896
Ponezumab	5	0.952 (0.304 to 2.988)	0.933	0%	0.816
**Fall**					
Bapineuzumab	6	0.994 (0.803 to 1.232)	0.958	0%	0.762
Bapineuzumab modified	5	1.965 (0.562 to 6.874)	0.29	0%	0.921
Donanemab	7	1.132 (0.619 to 2.071)	0.688	0%	0.817
Gantenerumab	3	0.924 (0.629 to 1.357)	0.687	0%	0.76
Ponezumab	5	0.814 (0.433 to 1.53)	0.524	0%	0.867
Solanezumab	10	0.941 (0.785 to 1.129)	0.514	0%	0.998
**Contusion**					
Donanemab	7	1.114 (0.331 to 3.744)	0.861	25.36%	0.235
Ponezumab	5	0.862 (0.388 to 1.919)	0.717	0%	0.616
Solanezumab	5	0.868 (0.576 to 1.309)	0.499	0%	1
**Skin laceration**					
Donanemab	6	2.204 (0.665 to 7.301)	0.196	0%	0.997
Solanezumab	5	0.982 (0.63 to 1.53)	0.936	0%	0.989
**Weight decreased**					
Bapineuzumab	3	1.412 (0.91 to 2.19)	0.124	0%	0.549
Bapineuzumab modified	5	1.907 (0.546 to 6.664)	0.312	0%	0.962
**Decreased appetite**					
Bapineuzumab modified	5	3.148 (0.97 to 10.216)	0.056	0%	0.801
Ponezumab	5	1.73 (0.54 to 5.544)	0.357	0%	0.874
**Musculoskeletal connective**					
Bapineuzumab	9	0.868 (0.424 to 1.777)	0.699	0%	0.706
**Back pain**					
Bapineuzumab	10	1.614 (1.027 to 2.539)	0.038	0%	0.916
Bapineuzumab modified	5	1.965 (0.562 to 6.874)	0.29	0%	0.921
Crenezumab	3	1.24 (0.511 to 3.009)	0.635	0%	0.997
Donanemab	6	2.204 (0.665 to 7.301)	0.196	0%	0.997
Gantenerumab	3	0.834 (0.558 to 1.247)	0.377	2.96%	0.357
Ponezumab	6	1.252 (0.543 to 2.889)	0.598	0%	0.649
Solanezumab	8	1.139 (0.891 to 1.456)	0.298	0%	0.997
**Pain**					
Bapineuzumab	6	0.553 (0.212 to 1.447)	0.228	0%	0.996
Solanezumab	7	1.392 (0.89 to 2.177)	0.147	0%	0.99
**Muscle spasms**					
Donanemab	6	2.204 (0.665 to 7.301)	0.196	0%	0.997
Solanezumab	6	1.101 (0.671 to 1.805)	0.704	0%	0.994
**Nervous system disorders**					
Bapineuzumab	8	1.176 (0.979 to 1.412)	0.083	23.08%	0.246
Donanemab	7	3.368 (1.49 to 7.612)	0.004	0%	0.974
Solanezumab	4	0.808 (0.713 to 0.916)	0.001	0%	0.499
**Headache**					
Bapineuzumab	13	1.064 (0.902 to 1.256)	0.463	0%	0.948
Bapineuzumab modified	5	1.956 (0.559 to 6.842)	0.293	0%	0.929
Donanemab	7	2.644 (0.838 to 8.345)	0.097	44.89%	0.092
Ponezumab	6	0.542 (0.297 to 0.991)	0.047	0%	0.744
Solanezumab	8	0.98 (0.787 to 1.22)	0.854	0%	0.802
**Dizziness**					
Bapineuzumab	10	1.054 (0.832 to 1.334)	0.665	0%	0.972
Bapineuzumab modified	5	1.078 (0.38 to 3.057)	0.888	0%	0.914
Donanemab	7	0.988 (0.502 to 1.943)	0.972	0%	0.823
Ponezumab	5	0.573 (0.237 to 1.385)	0.216	0%	0.911
Solanezumab					
**Intracranial hemorrhage**					
Bapineuzumab	11	1.455 (0.807 to 2.626)	0.213	38.18%	0.095
Donanemab	6	4.966 (1.68 to 10.674)	0.004	0.00%	0.976
Ponezumab	5	0.655 (0.332 to 1.289)	0.22	1.73%	0.397
**Syncope**					
Bapineuzumab	4	1.174 (0.734 to 1.876)	0.503	0.00%	0.47
Solanezumab	6	0.861 (0.585 to 1.267)	0.448	0.00%	0.981
**Psychiatric**					
Bapineuzumab	11	0.975 (0.847 to 1.122)	0.724	0.00%	0.853
Ponezumab	5	1.907 (0.72 to 5.05)	0.194	0.00%	0.902
Solanezumab	6	0.942 (0.814 to 1.092)	0.428	0.00%	0.711
**Depression**					
Bapineuzumab	10	0.821 (0.663 to 1.017)	0.071	0.00%	0.449
Crenezumab	3	0.811 (0.316 to 2.082)	0.663	0.00%	0.748
Ponezumab	7	0.905 (0.361 to 2.269)	0.832	0.00%	0.731
Solanezumab	3	1.017 (0.778 to 1.331)	0.9	0.00%	0.773
**Agitation**					
Bapineuzumab	7	1.016 (0.811 to 1.272)	0.892	21.29%	0.267
Donanemab	6	2.204 (0.665 to 7.301)	0.196	0.00%	0.997
Ponezumab	5	1.087 (0.476 to 2.479)	0.843	0.00%	0.748
Solanezumab	6	1.066 (0.786 to 1.445)	0.681	0.00%	0.988
**Irritability**					
Donanemab	6	2.204 (0.665 to 7.301)	0.196	0.00%	0.997
Ponezumab	7	0.576 (0.228 to 1.454)	0.243	0.00%	0.924
**Anxiety**					
Bapineuzumab	11	1.453 (0.784 to 2.694)	0.236	86.42%	<0.001
Bapineuzumab modified	5	0.93 (0.314 to 2.753)	0.896	0.00%	0.877
Crenezumab	3	4.238 (1.189 to 15.111)	0.026	0.00%	0.58
Donanemab	7	2.605 (1.001 to 6.783)	0.05	0.00%	0.997
Ponezumab	5	1.182 (0.525 to 2.663)	0.687	0.00%	0.759
Solanezumab	6	0.939 (0.73 to 1.208)	0.626	0.00%	0.999
**Insomnia**					
Bapineuzumab	5	1.306 (0.827 to 2.063)	0.252	0.00%	0.896
Ponezumab	5	0.753 (0.234 to 2.424)	0.635	19.71%	0.289
Solanezumab	3	1.013 (0.729 to 1.409)	0.938	0.00%	0.416
**Cough**					
Bapineuzumab	5	1.049 (0.681 to 1.615)	0.829	0%	0.636
Ponezumab	5	1.118 (0.486 to 2.572)	0.793	1.37%	0.399
Solanezumab	8	1.007 (0.813 to 1.246)	0.952	0%	0.774
**Skin**					
Bapineuzumab	4	1.544 (0.44 to 5.42)	0.498	0%	0.681
Donanemab	6	1.02 (0.354 to 2.936)	0.971	0%	0.994
Gantenerumab	3	1.336 (0.948 to 1.881)	0.098	0%	0.902
Solanezumab	7	1.05 (0.835 to 1.32)	0.677	0%	0.715
**Vascular**					
Bapineuzumab	3	1.01 (0.396 to 2.576)	0.983	0%	0.88
Solanezumab	5	0.804 (0.603 to 1.073)	0.138	0%	0.999
**Hypertension**					
Bapineuzumab	7	1.172 (0.49 to 2.802)	0.721	0%	0.991
Ponezumab	7	1.711 (0.703 to 4.164)	0.236	0%	0.813
**Renal and urinary disorders**					
Donanemab	6	2.999 (0.939 to 9.578)	0.064	0%	0.945
**Amyloid-related imaging abnormalities**					
Bapineuzumab	12	9.738 (2.061 to 46.003)	0.004	90.45%	<0.001
Bapineuzumab modified	5	1.907 (0.546 to 6.664)	0.312	0%	0.962
Donanemab	7	3.063 (3.525 to 23.3)	<0.001	0%	0.546
Gantenerumab	3	13.145 (5.215 to 33.136)	<0.001	0%	0.724
Solanezumab	3	1.224 (0.799 to 1.875)	0.354	0%	0.54

OR, odds ratio; 95% CI, 95% confidence interval.

Regarding solanezumab, there was a significantly reduced risk for nervous system disorders (OR: 0.808; 95% CI: 0.713–0.916). For ponezumab, the risk of headache was reduced significantly (OR: 0.542; 95% CI: 0.297–0.991). In contrast, gantenerumab was associated with a significantly increased risk of amyloid-related imaging abnormalities (OR: 13.145; 95% CI: 5.215–33.136).

Rare adverse events associated with monoclonal antibodies against Aβ *vis-à-vis* placebo in the treatment of mild or moderate AD are presented in Supplementary Table 2.

## Discussion

The aim of this meta-analysis was to summarize data on the effectiveness and safety of monoclonal antibodies against Aβ *vis-à-vis* placebo in the treatment of mild or moderate AD. It is noteworthy that monoclonal antibodies against Aβ as a whole can effectively improve instrumental activities of daily life based on CDR-SB scores. Moreover, analysis of individual antibodies revealed that bapineuzumab can improve cognition and function, as well as activities of daily life, yet it also triggers the occurrence of serious adverse events. To the best of our knowledge, this is the largest meta-analysis thus far that has synthesized data on monoclonal antibodies against Aβ compared with placebo for mild or moderate AD.

The deposit of extracellular Aβ plaques is a key feature of AD, and mounting evidence indicates that aberrant Aβ production or clearance is a potential harbinger in the pathogenesis of AD (48). Immunotherapy with monoclonal antibodies is increasingly identified as an effective therapeutic regime against AD, and dozens of clinical trials have been undertaken to explore the effectiveness and safety of monoclonal antibodies against Aβ in patients with AD (11, 49, 50). However, the results of these trials are not often reproducible. For example, Doody et al. in a multicenter, randomized, placebo-controlled trial demonstrated a marginally significant increase in MMSE scores in favor of donepezil (51), and contrastingly, Rinne et al. found that bapineuzumab exerted an unfavorable effect on MMSE scores (21). The reasons for these inconsistencies are likely several-fold. One reason might be related to sample sizes, because the magnitude of changes in instrumental scores between interventions is small in most cases. Another reason is probably due to the diverse types of monoclonal antibodies against Aβ, in view of the different targeted Aβ epitopes (31, 36, 52–56). A third reason rests with the differences in demographic and clinical characteristics, as well as genetic undergrounds across trials. Fortunately, meta-analysis offers a rational and helpful approach to dealing with inconsistencies from many studies of the same research topic. With the help of this approach and based on 29 articles and 21,383 participants, we interestingly found that monoclonal antibodies against Aβ as a whole can effectively improve instrumental activities of daily life based on CDR-SB scores in patients with mild or moderate AD, in line with the observations of many clinical trials (26, 36–38).

In addition, we explored the effectiveness and safety of individual monoclonal antibodies against Aβ in patients with AD. Because of the limited number of eligible trials, statistical significance was merely identified for bapineuzumab, an antibody targeted against the N-terminus of Aβ as reflected by MMSE and DAD scores, which can not only improve cognition and function but also enhance activities of daily life, as reflected by CDR-SB scores in terms of effectiveness. Simultaneously, the administration of bapineuzumab was associated with the development of serious adverse events. We agree that the safety profile is paramount, and the long-term benefits and risks of bapineuzumab treatment for mild or moderate AD are not yet known (25, 41). However, we here express concerns that such warnings may discourage patients and their families from choosing bapineuzumab in practice. From another aspect, Aβ might not be the best treatment target in patients with mild or moderate AD, or monoclonal antibodies against Aβ cannot remove an important species of Aβ that plays a contributing role in the pathogenesis of AD (37). Nevertheless, we agree that more large-scale clinical trials with long-term extended follow-ups are warranted to unveil the full potential of monoclonal antibodies against Aβ in AD.

In addition to the clear strengths of this meta-analysis, including the largest sample size, comprehensive analyses, and solid observations, several limitations should be acknowledged. First, only clinical trials published in English were retrieved, which leaves selection bias an open question, as some excellent trials may be published in other languages. However, explorations on publication bias revealed a low probability. Second, the power to detect significance in some subgroups was limited, and between-trial heterogeneity cannot be totally accounted for. Third, only the effectiveness and safety of monoclonal antibodies against Aβ *vis-à-vis* placebo were examined in the current meta-analysis, and comparison between other classes of drugs targeting AD will be addressed in the future. Fourth, definitions of adverse effects evaluated in this meta-analysis differed across trials, and caution is needed when interpreting the safety profiles of monoclonal antibodies.

Taken together, our findings indicate that monoclonal antibodies against Aβ as a whole can effectively improve instrumental activities of daily life based on CDR-SB scores in mild or moderate AD. Individually, bapineuzumab can improve cognition and function, as well as activities of daily life, yet it also triggers the occurrence of serious adverse events. Further functional investigations on the molecular mechanisms of monoclonal antibodies against Aβ, in particular, bapineuzumab, in the pathophysiology of AD.

## Data availability statement

The original contributions presented in the study are included in the article/Supplementary material, further inquiries can be directed to the corresponding authors.

## Ethics statement

The studies involving human participants were reviewed and approved by Ethics Committees of all institutes or hospitals involved in this meta-analysis. The patients/participants provided their written informed consent to participate in this study.

## Author contributions

WN and XD planned and designed the study. WN directed its implementation. YH and MD contributed to data acquisition and conducted statistical analyses. YH, MD, YS, and XD had access to all raw data. YH and WN wrote the manuscript. All authors have read and approved the final manuscript prior to submission.
