# Supplementary material for: Effectiveness and safety of monoclonal antibodies against amyloid-beta vis-à-vis placebo in mild or moderate Alzheimer's disease
Source: Front Neurol. 2023 Mar 15;14:1147757. doi: 10.3389/fneur.2023.1147757 (PMC10050585; doi:10.3389/fneur.2023.1147757)
Supplement: Supplementary file 1 [file Data_Sheet_1.PDF]

Supplementary Table 1.

PRISMA 2009 Checklist

Section/topic	#	Checklist item	Reported on page #
**TITLE**			
Title	1	Identify the report as a systematic review, meta-analysis, or both.	1
**ABSTRACT**			
Structured summary	2	Provide a structured summary including, as applicable: background; objectives; data sources; study eligibility criteria, participants, and interventions; study appraisal and synthesis methods; results; limitations; conclusions and implications of key findings; systematic review registration number.	2
**INTRODUCTION**			
Rationale	3	Describe the rationale for the review in the context of what is already known.	3-4
Objectives	4	Provide an explicit statement of questions being addressed with reference to participants, interventions, comparisons, outcomes, and study design (PICOS).	5
**METHODS**			
Protocol and registration	5	Indicate if a review protocol exists, if and where it can be accessed (e.g., Web address), and, if available, provide registration information including registration number.	5
Eligibility criteria	6	Specify study characteristics (e.g., PICOS, length of follow-up) and report characteristics (e.g., years considered, language, publication status) used as criteria for eligibility, giving rationale.	6
Information sources	7	Describe all information sources (e.g., databases with dates of coverage, contact with study authors to identify additional studies) in the search and date last searched.	5
Search	8	Present full electronic search strategy for at least one database, including any limits used, such that it could be repeated.	5
Study selection	9	State the process for selecting studies (i.e., screening, eligibility, included in systematic review, and, if applicable, included in the meta-analysis).	5
Data collection process	10	Describe method of data extraction from reports (e.g., piloted forms, independently, in duplicate) and any processes for obtaining and confirming data from investigators.	7
Data items	11	List and define all variables for which data were sought (e.g., PICOS, funding sources) and any assumptions and simplifications made.	7
Risk of bias in individual studies	12	Describe methods used for assessing risk of bias of individual studies (including specification of whether this was done at the study or outcome level), and how this information is to be used in any data synthesis.	7-8
Summary measures	13	State the principal summary measures (e.g., risk ratio, difference in means).	7-8
Synthesis of results	14	Describe the methods of handling data and combining results of studies, if done, including measures of consistency (e.g., I^2) for each meta-analysis.	7-8

PRISMA 2009 Checklist

Section/topic	#	Checklist item	Reported on page #
Risk of bias across studies	15	Specify any assessment of risk of bias that may affect the cumulative evidence (e.g., publication bias, selective reporting within studies).	7-8
Additional analyses	16	Describe methods of additional analyses (e.g., sensitivity or subgroup analyses, meta-regression), if done, indicating which were pre-specified.	7-8
**RESULTS**			
Study selection	17	Give numbers of studies screened, assessed for eligibility, and included in the review, with reasons for exclusions at each stage, ideally with a flow diagram.	Figure 1
Study characteristics	18	For each study, present characteristics for which data were extracted (e.g., study size, PICOS, follow-up period) and provide the citations.	9-10
Risk of bias within studies	19	Present data on risk of bias of each study and, if available, any outcome level assessment (see item 12).	10-11
Results of individual studies	20	For all outcomes considered (benefits or harms), present, for each study: (a) simple summary data for each intervention group (b) effect estimates and confidence intervals, ideally with a forest plot.	Figure 2, Figure 3
Synthesis of results	21	Present results of each meta-analysis done, including confidence intervals and measures of consistency.	10-11, Figure 2, Figure 3
Risk of bias across studies	22	Present results of any assessment of risk of bias across studies (see Item 15).	Figure 3
Additional analysis	23	Give results of additional analyses, if done (e.g., sensitivity or subgroup analyses, meta-regression [see Item 16]).	12-13
**DISCUSSION**			
Summary of evidence	24	Summarize the main findings including the strength of evidence for each main outcome; consider their relevance to key groups (e.g., healthcare providers, users, and policy makers).	13-15
Limitations	25	Discuss limitations at study and outcome level (e.g., risk of bias), and at review-level (e.g., incomplete retrieval of identified research, reporting bias).	15-16
Conclusions	26	Provide a general interpretation of the results in the context of other evidence, and implications for future research.	16
**FUNDING**			
Funding	27	Describe sources of funding for the systematic review and other support (e.g., supply of data); role of funders for the systematic review.	No

From: Moher D, Liberati A, Tetzlaff J, Altman DG, The PRISMA Group (2009). Preferred Reporting Items for Systematic Reviews and Meta-Analyses: The PRISMA Statement. PLoS Med 6(6): e1000097. doi:10.1371/journal.pmed1000097

For more information, visit: **www.prisma-statement.org**.

Supplementary Figure 1. Cumulative analyses of four assessment scales for monoclonal antibodies against Aβ vis- à-vis placebo in the treatment of Alzheimer's disease. Abbreviations: MMSE, Mini-Mental State Examination; ADAS-Cog, Cognitive Subscale; DAD, Disability Assessment for Dementia; CDR-SB, Clinical Dementia Rating Scale-Sum of Boxes; SMD, standardized mean difference; 95% CI, 95% confidence interval.

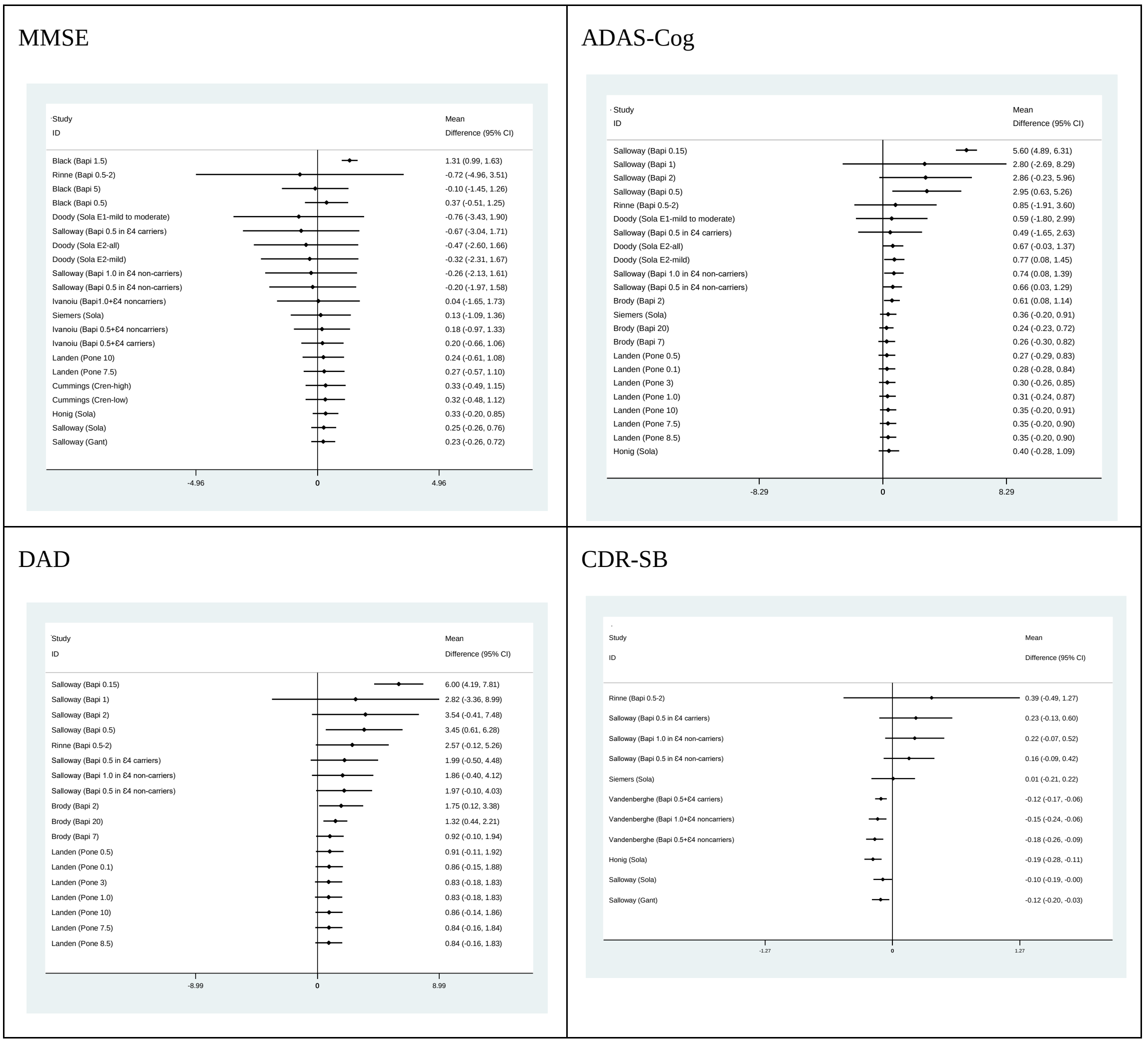

Supplementary Figure 2. Influential analyses of four assessment scales for monoclonal antibodies against Aβ vis- à-vis placebo in the treatment of Alzheimer’s disease. Abbreviations: MMSE, Mini-Mental State Examination; ADAS-Cog, Cognitive Subscale; DAD, Disability Assessment for Dementia; CDR-SB, Clinical Dementia Rating Scale-Sum of Boxes; SMD, standardized mean difference; 95% CI, 95% confidence interval.

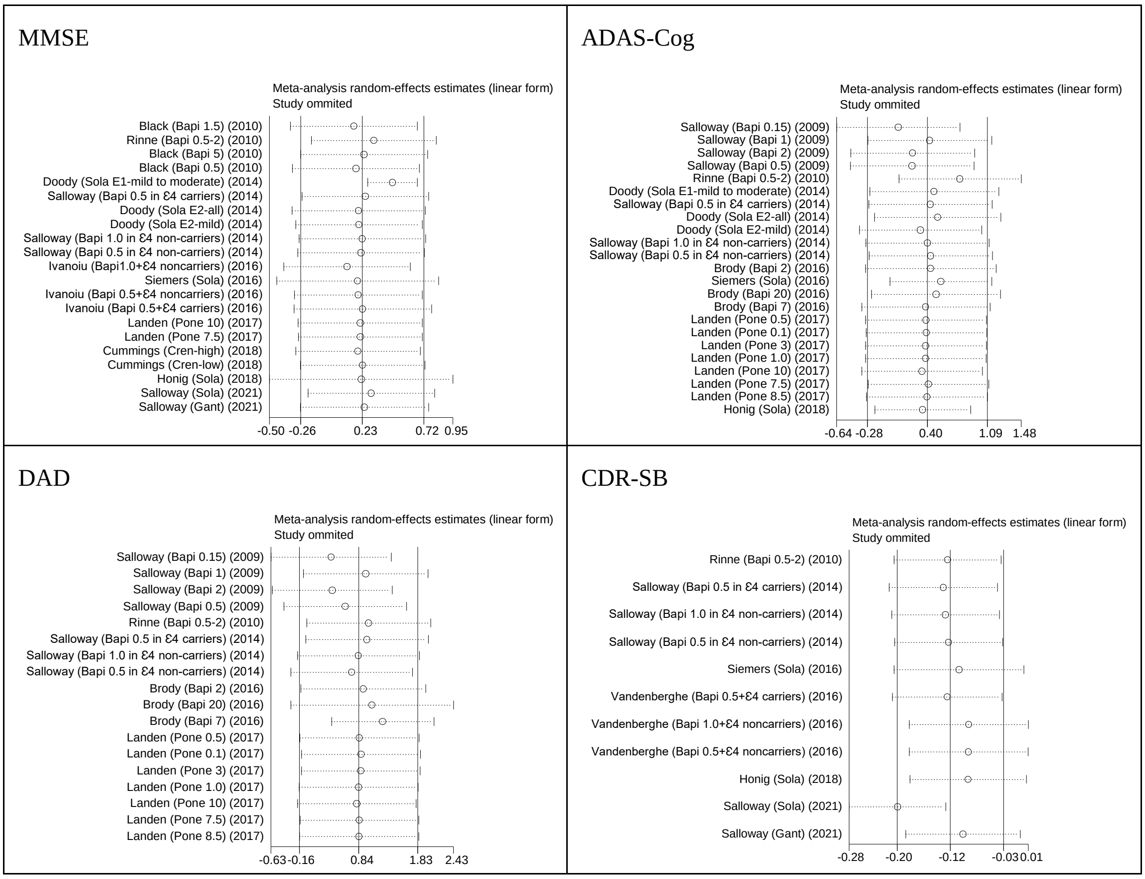

Supplementary Table 2. Rare adverse events associated with monoclonal antibodies against Aβ in the treatment of Alzheimer's disease.

Adverse events	Studies (n)	OR (95% CI)	P	I^2	P for heterogeneity
**Cardiac**					
Bapineuzumab	3	2.369 (1.099-5.109)	0.028	0%	0.785
Gantenerumab	2	1.001 (0.658-1.524)	0.995	0%	0.747
Solanezumab	3	1.037 (0.562-1.915)	0.908	72.15%	0.028
**Ear labyrinth**					
Bapineuzumab	3	1.182 (0.238-5.866)	0.838	0%	0.884
Gantenerumab	2	1.289 (0.728-2.285)	0.384	0%	0.854
**By cataracts**					
Bapineuzumab	5	1.708 (0.488-5.979)	0.402	0%	0.994
Gantenerumab	2	0.978 (0.617-1.549)	0.923	0%	0.355
Solanezumab	2	1.048 (0.785-1.4)	0.75	0%	0.753
**Gastrointestinal**					
Bapineuzumab	4	2.065 (0.834-5.112)	0.117	50.65%	0.108
Gantenerumab	2	0.972 (0.734-1.287)	0.844	0%	0.906
Solanezumab	2	0.99 (0.839-1.169)	0.907	16.91%	0.273
**Abdominal pain**					
Bapineuzumab	6	0.746 (0.247-2.26)	0.605	0%	0.964
**General administration**					
Bapineuzumab	3	0.372 (0.098-1.413)	0.147	0%	0.895
Solanezumab	2	0.947 (0.781-1.147)	0.575	0%	0.807
**Asthenia**					
Bapineuzumab	6	0.614 (0.214-1.761)	0.364	0%	0.998
Solanezumab	2	0.634 (0.347-1.158)	0.138	0%	0.976
**Influenza**					
Gantenerumab	2	1.084 (0.627-1.876)	0.772	0%	0.728
**Bronchitis**					
Bapineuzumab	3	1.182 (0.238-5.866)	0.838	0%	0.884
Gantenerumab	3	1.303 (0.758-2.242)	0.338	0%	0.716
**Nutrition disorders**					
Bapineuzumab	3	1.311 (0.589-2.918)	0.507	0%	0.588
Gantenerumab	2	0.979 (0.637-1.505)	0.924	0%	0.663
**Insufficient**					
Bapineuzumab	2	0.963 (0.594-1.561)	0.88	0%	0.335
Gantenerumab	2	0.687 (0.417-1.133)	0.142	0%	0.497
Solanezumab	2	0.786 (0.526-1.174)	0.24	0%	0.921
**Musculoskeletal pain**					
Solanezumab	2	1.003 (0.577-1.743)	0.993	0%	0.806
**Osteoarthritis**					
Bapineuzumab	3	0.751 (0.176-3.201)	0.699	0%	0.995
**Gait disturbance**					
Bapineuzumab	2	1.882 (0.335-10.583)	0.473	0.00%	0.346
Bapineuzumab modified	5	1.956 (0.559-6.842)	0.293	0.00%	0.929
**Muscle spasm**					
Bapineuzumab	7	1.261 (0.506-3.144)	0.619	0.00%	0.683

**Anxiety**					
Donanemab	6	2.611 (0.81-8.423)	0.108	0.00%	0.988
**Paranoia**					
Bapineuzumab	3	2.551 (0.596-10.91)	0.207	0.00%	0.769
Donanemab	6	2.204 (0.665-7.301)	0.196	0.00%	0.997
**Aggression**					
Bapineuzumab	3	1.024 (0.307-3.408)	0.97	0%	0.911
Ponezumab	5	1.56 (0.481-5.051)	0.459	0%	0.954
**Pulmonary embolism**					
Bapineuzumab	4	1.311 (0.345-4.977)	0.691	0%	0.991
**Deep vein thrombosis**					
Bapineuzumab	7	1.145 (0.539-2.433)	0.724	0%	0.726
**Neoplasms**					
Bapineuzumab	3	0.675 (0.277-1.644)	0.387	50.46%	0.133
Gantenerumab	2	0.923 (0.58-1.47)	0.737	0%	0.479

Abbreviations: OR, odds ratio; 95% CI, 95% confidence interval.
